# Nuclear Weapons and Neglected Diseases: The “Ten-Thousand-to-One Gap”

**DOI:** 10.1371/journal.pntd.0000680

**Published:** 2010-04-27

**Authors:** Peter J. Hotez

**Affiliations:** Sabin Vaccine Institute and Department of Microbiology, Immunology, and Tropical Medicine, The George Washington University, Washington, D. C., United States of America

## Introduction

Together, the world's eight acknowledged nuclear powers—the United States (US), Russia, United Kingdom (UK), France, China, India, Pakistan, and the Democratic People's Republic of Korea (North Korea)—have amassed an arsenal of almost 30,000 nuclear weapons since 1945. In addition, Israel is believed to be a nuclear power while Iran (and possibly Syria as well) is also suspected of developing nuclear weapons. Despite the technological sophistication that has enabled the 11 nuclear weapons states to produce and deliver nuclear bombs, most of these nations simultaneously also suffer from high internal rates of poverty and endemic neglected diseases. They include high prevalence rates of neglected tropical diseases in India, China, Pakistan, Iran, and Syria, and related neglected infections of poverty in the US and Europe. Indeed, the 11 nuclear weapons states together account for up to one-half of the global disease burden from all neglected diseases. However, for a tiny fraction (less than 1/10,000^th^) of the costs of producing and maintaining a nuclear arsenal the 11 nuclear powers could eliminate most of their neglected diseases and engage in joint neglected disease research and development efforts that help to reduce international tensions and promote world peace.

**Figure 1:**
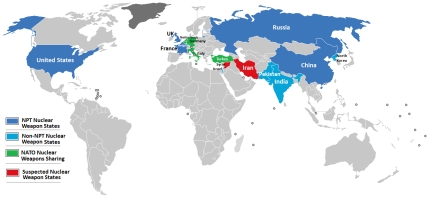
Map of countries with nuclear weapons. NPT, nuclear nonproliferation treaty. Source: http://commons.wikimedia.org/wiki/File:Nuclear_weapons.png.

Shown in Table 1 and Figure 1 are the 11 established and suspected global nuclear powers. Following the development and deployment of the atomic bomb by the US in 1945 (at an estimated cost of US$20 billion), Russia became the second nuclear power in 1949, and in every decade since then at least one new country has joined the nuclear club [1], [2]. In addition three countries, South Africa, Argentina, and Brazil, began active nuclear weapons programs, but subsequently abandoned them by mutual treaty [1]. Today, only the first five nations to produce nuclear weapons,,the US, Russia, UK, France, and China, have signed the nuclear nonproliferation treaty [1]. The costs to maintain these nuclear arsenals are staggering. According to the Brookings Institution, which in 1998 published their US Nuclear Weapons Cost Study Project, the US alone spent $35 billion that year on nuclear weapons technology [2]. Further estimates indicate that the US may have spent more than $5.5 trillion in developing their nuclear arsenal, while France has invested approximately $1.5 trillion [3]. Although the data are unavailable, the costs for other nuclear weapon states are believed to be similar [3]. Therefore it is likely that the 11 nuclear weapons states together have invested at least $10 trillion on weapons production and maintenance.

**Table 1:** Estimated number of nuclear weapons by Country, 1945–2008.

Country	Estimated number of nuclear weapons	Date first test conducted	Member of Nuclear Non-Proliferation Treaty?
United States	10,000	1945	Yes
USSR/Russia	16,000	1949	Yes
United Kingdom	200	1952	Yes
France	350	1960	Yes
China	410	1964	Yes
India	75–110	1974	No
Israel	100–200	1979?	No
Pakistan	60	1998	No
DPR Korea (North Korea)	<10	2006	No
Iran	Suspected nuclear weapons state	Not determined	No
Syria	Suspected nuclear weapons state	Not determined	No

Sources: [1] and http://en.wikipedia.org/wiki/List_of_states_with_nuclear_weapons.

Despite this massive expenditure, each of the 11 nuclear weapons states, with the possible exception of the U.K., also suffers from high rates of neglected tropical diseases (and related neglected infections of poverty), defined as chronic and debilitating parasitic and other infectious diseases that occur in association with extreme poverty [4]. In addition to their health effects, the neglected tropical diseases also cause poverty through their ability to impair child physical and intellectual development, pregnancy outcomes, and worker productivity, while simultaneously promoting conflict and war through their agriculturally and socially destabilizing effects [4], [5]. Although it is common to think of neglected diseases as confined to low-income countries in sub-Saharan Africa, Southeast Asia, and Latin America, as shown in Table 2 these infections also exhibit a high prevalence in middle-income countries such as China, India, Pakistan, North Korea, Iran, and Syria, as well as in selected areas of poverty found in the US, Russia, and Eastern Europe [6]. Indeed, with the possible exceptions of the UK, high neglected disease burdens are present in all of the nuclear weapons states, particularly the helminth infections, leishmaniasis and Chagas disease, toxoplasmosis, and trachoma.

**Table 2:** The major neglected tropical diseases or neglected infection of poverty endemic to countries with nuclear weapons.

Country	Neglected infection	Neglected infection	Neglected infection	Neglected infection	Neglected infection	Reference
	(Est. Cases)	(Est. Cases)	(Est. Cases)	(Est. Cases)	(Est. Cases)	
United States	Toxocariasis1–3 million	Trichomoniasis1 million	Chagas disease<1 million	Cysticercosis<0.2 million	Strongyloidiasis0.1 million	[10], [29]
USSR/Russia	ToxoplasmosisPrevalence ND	Opisthorchiasis12.5 million at risk	Congenital syphilisIncidence 170/100,000 in 1996			[13], [16] [29], [32]
United Kingdom	ToxocariasisPrevalence ND	ToxoplasmosisPrevalence ND				[29]
France	Toxoplasmosis59%seroprevalence in pregnancy	LeishmaniasisIncidence 0.02–0.19/100,000	Chagas diseasePrevalence ND	StrongyloidiasisPrevalence ND		[11], [21], [22], [28] [29]
China	Ascariasis86 million	Hookworm39 million	Trichuriasis29 million	Trachoma27 million	Paragonimiasis Clonorchiasis13 million	[9], [13], [14] [30]
India	Ascariasis140 million	Trichuriasis73 million	Hookworm71 million	Lymphatic filariasis30 million	Trachoma1 millionToxoplasmosis12–45%seroprevalenceIn pregnancyLeishmaniasis0.3 million	[8], [12], [17], [29] [30]
Israel	LeishmaniasisIncidence 0.13–7/100,000					[27]
Pakistan	Ascariaisis21 million	Hookworm2 million	Trichuriasis1.5 million	Trachoma0.3 million	LeishmaniasisPrevalence ND	[8], [18], [26], [30]
DPR Korea(North Korea)	Ascariasis8 million	Hookworm1 million	Trichuriasis0.2 million	ClonorchiasisPrevalence ND		[8], [15]
Iran	Ascariasis5 million	Trichuriasis1.6 million	Hookworm0.4 million	Toxoplasmosis29–64% seroprevalence in pregnancy or women	LeishmaniasisPrevalence NDTrachoma15,000	[8], [19], [24], [25], [29] [30]
Syria	LeishmaniasisPrevalence ND					[20], [23]

ND, not determined.

## Helminthic Neglected Infections

The four major soil-transmitted helminth infections of humans include ascariasis (roundworm), trichuriasis (whipworm), hookworm infection, and strongyloidiasis (threadworm) [7]. These intestinal worm infections represent the most common neglected tropical diseases of humans living in poverty [7], [8]. Of the estimated 800 million ascariasis infections found predominantly in low- and middle-income countries [7], approximately one-third of the cases occur in nuclear weapons states including India (140 million), China (86 million), North Korea (8 million), Pakistan (7 million), and Iran (5 million) [8], [9]. These nations also account for 20% of the world's cases of hookworm infection, which is associated with anemia and extreme poverty resulting from impairments in child development and cognition, maternal morbidity during pregnancy, and diminished agricultural worker productivity [4], [7], [8], [9]. Trichuriasis and strongyloidiasis are endemic to these countries as well, and the US and France have pockets of endemic strongyloidiasis in, respectively, Appalachia and in Region Midi-Pyrenees in the Southwest [10], [11]. In the US and elsewhere, toxocariasis is a soil-transmitted helminth zoonosis associated with larval migrans syndromes, asthma, and developmental delays in up to 3 million African Americans living in poverty [10].

India accounts for roughly one-quarter of the world's 120 million cases of lymphatic filariasis, a disfiguring and stigmatizing vector-borne infection associated with elephantiasis [12], while China accounts for most of the world's food-borne trematode infections, including 13 million cases of clonorchiasis (liver fluke infection associated with liver fibrosis and bile duct cancer) and paragonimiasis (lung fluke infection associated with hemoptysis and other pulmonary disorders) [9], [13], [14]. Clonorchiasis is also endemic to North Korea [15], while a related liver fluke infection known as opisthorchiasis is highly endemic to Russia where an estimated 12.5 million people are at risk of infection, and in some parts of Siberia up to 95% of the population is infected [13], [16]. Up to 169,000 cases of cysticercosis occur among Hispanic Americans in the US [10].

## Leishmaniasis and Other Protozoan Neglected Infections

Leishmaniasis is a serious sandfly-transmitted neglected tropical disease endemic to the Asian and Middle Eastern nuclear states where it is associated with extreme poverty [4], [17]. The visceral form (caused by *Leishmania donovani* in Asia and the Middle East) is the most severe and is associated with profound pancytopenia, hepatosplenomegaly, and failure to thrive when it occurs in childhood. Annually, there are approximately 500,000 new cases of visceral leishmaniasis worldwide with more than one-half of these cases (270,000) occurring in India alone [17]. Most of the Indian cases occur in the impoverished state of Bihar [17], while in Pakistan visceral leishmaniasis is endemic to Azad Jammu and Kashmir, on the Indian border [18]. Visceral leishmaniasis is also endemic to Iran and Syria [19], [20], and it occurs in southern France where it is a zoonosis caused by *L. infantum* with dogs as the major animal reservior, and recognized as an opportunistic infection with patients with HIV/AIDS [21], [22]. In most of the countries where visceral leishmaniasis is found [22]–[26], including Israel [27], the cutaneous form is also present and caused by either *L. major* or *L. tropica*. Cutaneous leishmaniasis is disfiguring and highly stigmatizing, especially for young women [4]. In the US, cutaneous leishmaniasis caused by *L. mexicana* and possibly other species has emerged along the border with Mexico [10]. Up to one million cases of Chagas disease are found in the US as well, mostly among Hispanic American immigrants, but there is also evidence of Chagas disease transmission within the borders of the US [10]. Chagas disease is also found in France [28]. Toxoplasmosis is an important congenital protozoan infection that results in blindness and profound mental disabilities found in all of the nuclear weapons states, but especially the US and France [29].

## Bacterial Neglected Diseases

Almost one-half of the world's 60–80 million trachoma cases occur in nuclear weapons states, with China having the highest number of cases of any nation [30]. Similarly, India has the highest number of leprosy cases, with more than one-half of the new infections that occur annually in the world [31]. Following the collapse of the former Soviet Union in 1991, as a result of the disintegration of the state-run health care system and other political and socioeconomic changes, the rate of congenital syphilis rose at a rate 34 times higher than was seen in Western Europe [32].

## Summary of Neglected Diseases in Nuclear Weapons States

Although the world's nuclear states have up to one-third of the world's cases of soil-transmitted helminth infections, more than one-half of the world's new cases of leishmaniasis and leprosy, and approximately one-half of the global disease burden of trachoma, they have chosen to devote their major resources to weapons production instead of neglected disease control [33]. For example, India allocates only $0.40 per individual per year for treatment of its population at risk for visceral leishmaniasis [17], while the projected US government annual budget in 2010 for global neglected tropical disease control is expected to be $65 million (part of a larger $350 million U.S. commitment), roughly 1,000 times less than its annual nuclear weapons budget [34]. The UK government has committed approximately £50 million to neglected tropical disease control [35]. A distinction can be made between states that can afford more nuclear weapons (i.e., US, UK, France) and those for which social spending must be curtailed to pursue nuclear ambitions (i.e., India, Pakistan, China). In terms of research and development (R&D) funding the George Institute estimates that the global budget for neglected tropical diseases is approximately $400 million including only $125 million for leishmaniasis and trypanosome infections, $80 million for dengue, $50 million for all helminth infections, and less than $10 million each for trachoma, leprosy and Buruli ulcer, trachoma, and typhoid/paratyphoid [36]. Together, all of the costs for both neglected disease control and R&D come close to $1 billion, or roughly less than 1/10,000^th^ of the estimated $10 trillion committed for nuclear weapons.

While some may argue that the trillions of dollars spent on nuclear weapons may have served as a successful deterrent for an all-out US-Soviet, a Sino-Soviet, or an Indo-Pakistan conflict and was therefore an actual investment in peace, imagine a world in which the nuclear weapons states increased their neglected disease control budgets more than 10-fold thereby, reducing the global nuclear weapon–to–neglected disease gap to 1,000 to 1. Ten billion dollars devoted to neglected diseases would be sufficient to effect large-scale control or elimination for most of the high-prevalence neglected tropical diseases in the 56 nations where five or more of these conditions are endemic [37]–[38]. Among the nuclear weapons states these 56 nations include India and China, although sufficient funds would remain to tackle neglected infections among pockets of poverty in the remaining wealthy nine nuclear countries. In addition to the enormous health impact of controlling or eliminating conditions with a combined disease burden that exceeds malaria or tuberculosis, because the neglected tropical diseases also contribute to poverty, reducing this disease burden would help reduce poverty and therefore help achieve the Millennium Development Goals [38]. Neglected tropical disease control and elimination would also help stabilize and build nations, reduce civil strife and international tensions, and contribute to world peace [5], [38]. Control of neglected infections of poverty would eliminate an important group of health disparities in the US and Europe [6], while simultaneously providing a vehicle by which nations such as India and China would attain new status as global leaders.

Scientific and technical cooperation between nuclear weapons states should also be enhanced in order to improve sanitation and water quality through collaborative ventures in engineering and urban planning, and to promote R&D for new drugs and vaccines to combat neglected tropical diseases [39], [40]. Similar “vaccine diplomacy” efforts between the US and Soviet Union during the Cold War led to the successful development, testing, and distribution of the oral polio vaccine and the smallpox vaccine [41], [42] Greater efforts are needed to engage leaders of the nuclear weapons states in a frank dialogue about reallocation of resources toward public health and scientific pursuits for neglected tropical disease R&D and control. Several possibilities exist for such engagement. Conferences are held every five years to review the operation of the nuclear nonprofileration treaty [43], and time should be set aside at these meetings for purposes of disease control and biomedical research. In 2009, the first BRIC (Brazil, Russia, India, China) summit took place in Russia [44], and in the future this vehicle could also be used to establish a meaningful dialogue on neglected diseases. In the coming decade, engaging the nuclear powers on neglected disease R&D and global implementation efforts would help to achieve Millennium Development Goals and would represent a significant diplomatic victory for the world.
